# *Lophorrhinidesmuellerae* (Coleoptera, Scarabaeidae, Cetoniinae): a new genus and species from southern Tanzania

**DOI:** 10.3897/zookeys.833.31502

**Published:** 2019-03-26

**Authors:** Renzo Perissinotto, Lynette Clennell, Gerhard Beinhundner

**Affiliations:** 1 School of Environmental Sciences, Nelson Mandela University, P.O. Box 77000, Port Elizabeth 6031, South Africa Nelson Mandela University Port Elizabeth South Africa; 2 Macau Anglican College, 109-117 Avenida Padre Tomas Pereira, Taipa-Macau, China Macau Anglican College Taipa-Macau China; 3 Am Steigberg 25, D-97502 Euerbach, Germany Unaffiliated Euerbach Germany

**Keywords:** Fruit chafers, *
Lophorrhina
*, East African Rift, Afrotropical region

## Abstract

A male cetoniine specimen recently submitted for identification from the Ditsong Museum of Natural History (Pretoria, South Africa) has been found to represent a yet unknown species. A review of the recently published book of Beinhundner (2017) has further revealed that one of the specimens mistakenly figured as *Lophorrhinadonckieri* Bourgoin, 1913 in that work is most likely the female of this new species. Analysis of the diagnostic characters of the genus *Lophorrhina* Westwood, 1842 shows that the new species differs in several key areas. In particular, the clypeal armature is virtually identical in both sexes, the male protibiae are not typically elongate and narrow as in all the members of *Lophorrhina*, but are remarkably more robust, laterally expanded and with a tridentate margin in both sexes, even though the third tooth in the female and the second and third teeth in the male are virtually obsolete. The general body shape in the new species is also more globose and lacks the typical deplanate and apically tapering elytra of the *Lophorrhina* males. These and other characters are, in our view, sufficient to justify the erection of a new genus, *Lophorrhinides***gen. n.**, to accommodate the new species, here described as *L.muellerae***sp. n.** The new genus is presumably a mountain specialist, as both known specimens were collected in the southern highlands of Tanzania, at Manow and Rungwe respectively.

## Introduction

A male cetoniine specimen from an old collection originating from “Deutsch Ost-Afrika” and submitted for identification in 2016 by the Ditsong Museum of Natural History (Pretoria, South Africa) has revealed unique characteristics, with affinities to *Lophorrhina* Westwood, 1842 and, to a lesser extent, genera such as *Anisorrhina* Westwood, 1842 and *Chlorocala* Kirby, 1828, particularly at the level of the parameres. A female specimen belonging to the same genus and species was also recognised among a series of photographs included under *Lophorrhinadonckieri* Bourgoin, 1913, after the publication of the monograph on the “Cetoniinae of Africa” by Beinhundner (2017; p. 990, fig. 18). This has made it possible to complete and substantiate the description of a new species.

The genus *Lophorrhina* Westwood, 1842 currently includes the synonymic genera *Chordodera* Burmeister, 1842, *Daedycorrhina* Bates, 1888 *Aphanesthes* Kolbe, 1892, and *Aphanochroa* Kolbe, 1893. It includes 13 described species, most of which are high altitude endemics of the Tanzanian mountains. The new species described here occurs close to the southern limit of the distribution range of *Lophorrhina* and exhibits several distinct characters that may reflect geographical isolation from the ancestral lineage. In particular, the general body shape is rather globose and not deplanate like in *Lophorrhina*, the scutellum is equilateral triangular and exhibits dense and long setae, while in *Lophorrhina* this is isosceles triangular and virtually asetose. The clypeal shape is also remarkably different to that of *Lophorrhina*, in that the horn is virtually obsolete in both sexes and the clypeal margins are laterally expanded to form a general shape broader but shorter than in *Lophorrhina*.

These and other differences highlighted in the description below make it impossible to include with confidence this species within any existing genera of the African cetoniines, thereby necessitating the erection of a new genus, *Lophorrhinides* gen. n. This adds to the already remarkable diversity observed for this beetle group in the Afrotropical region (excluding Madagascar), where 138 genera and more than 1000 species are currently recognised (Sakai and Nagai 1998, Beinhundner 2017).

## Materials and methods

The only two specimens currently known for this new genus and species were analysed after obtaining a loan from the Ditsong Museum of Natural History (TMSA, Pretoria, South Africa) and through direct access to the Private Collection of Gerhard Beinhundner (PCGB, Euerbach, Germany), respectively.

As in previous work, the description of morphological characters of this study follows the terminology used by Krikken (1984) and Holm and Marais (1992). Specimen total length and maximum width were measured using a Vernier calliper, from the anterior margin of the clypeus to the apex of the pygidium and at the widest point of the elytra, respectively.

Photos of the dorsal and ventral habitus were taken with a Nikon CoolPix S9700 and a Nikon CoolPix 990 digital camera with macro setting, while photos of the male genitalia were obtained using a Nikon DigitalSight DS-Fi2 camera attached to a Nikon SMZ25 dissecting microscope. The background was removed from the photos using Microsoft Word 2010 (Picture Tools) and Adobe Photoshop 7.0, in order to increase clarity of resolution. The Combine ZP Image Stacking Software by Alan Hadley (alan@micropics.org.uk) was used to obtain z-stacking composite images.

## Results

### Lophorrhinides gen. n.

Taxon classificationAnimaliaColeopteraScarabaeidae

http://zoobank.org/76FA4EC4-214F-499F-9C58-8823A8535E79

#### Type species

*Lophorrhinidesmuellerae* gen. et sp. n.

#### Diagnosis

The new genus is most closely related to *Lophorrhina* Westwood, 1842. It differs from this genus mainly by its generally dense pubescence on the dorsal surface (particularly well-developed in male), the fairly globose rather than deplanate body shape, the hemicircular rather than octagonal pronotal shape, the equilateral rather than isosceles triangular shape of its scutellum, the presence of reduced clypeal armature, which is similar in both sexes, and the reduction of the central horn on head frons to a tubercle. A more comprehensive comparison between the two genera is provided in Table 1 below.

**Table 1.** Comparison of diagnostic generic characters of *Lophorrhina* Westwood, 1842 versus *Lophorrhinides* gen. n. The list for *Lophorrhina* includes the key features highlighted in the original description of Westwood (1842) as well as those of the successive synonyms of *Chordodera* Burmeister, 1842, *Daedycorrhina* Bates, 1888, *Aphanesthes* Kolbe, 1892 and *Aphanochroa* Kolbe, 1893.

***Lophorrhina* Westwood, 1842**	***Lophorrhinides* gen. n.**
Clypeal horn flat to concave but truncated in male, drastically reduced in female to a slight elevation	Clypeal horn deeply concave, wide but short in both sexes, only slightly reduced in female compared to male
Presence of flattened, central horn at posterior margin of head frons, between eyes	Presence of central tubercle at posterior margin of head frons, between eyes
Scutellum virtually asetose, isosceles triangular in shape with width/height ratio ≤ 1	Scutellum with dense pilosity, equilateral triangular in shape with width/height ratio > 1
Tridentate protibia in both sexes, but all teeth obsolete in male	Tridentate protibiae in both sexes, but teeth 2-3 obsolete in male and tooth 3 drastically reduced in female
Tibiae and tarsi thin and elongate to hypertrophic in male (especially in prolegs, where tibiae are often arcuate)	Tibiae and tarsi of normal cetoniine length and thickness, with no visible sexual dimorphism
Mesometasternal lobe dilated into a short, round process and protruding forward	Mesometasternal lobe smoothly rounded and not expanded anteriorly or laterally
Body deplanate, with pronotum virtually octagonal and elytra tapering towards apex	Body substantially globose, with hemicircular pronotum exhibiting heavily sinuate posterior margin and elytra smoothly rounded at apex
Ornamentation well developed, with velutinous surface exhibiting longitudinal lines or stripes on pronotum and light-dark maculation on elytra	Ornamentation poorly developed, with pronotum exhibiting only very restricted orange maculation on antero-lateral margin and elytra with dark patches only on umbones and on and around sutural margins
Pilosity absent or drastically reduced on dorsal surface in both sexes	Body surface with dense orange pubescence, which is substantially reduced on female dorsum but still prominent on head and scutellum
Parameres with ventral lobes at apical half wider than dorsal ones and protruding laterally, dorsal lobes arcuate with lateral expansion near apex	Parameres with ventral lobes wider than dorsal ones and protruding laterally, dorsal lobes arcuate with longitudinal groove depression at middle and lateral expansion near apex

#### Derivatio nominis

The new genus name clearly refers to its close relationship with the sister genus *Lophorrhina* Westwood, 1842.

### Lophorrhinides muellerae sp. n.

Taxon classificationAnimaliaColeopteraScarabaeidae

http://zoobank.org/89671880-3FD5-4B7D-80C8-D6E838C13938

Fig. 1A–F

#### Type material.

Holotype male: Manow DO Afr, Sammlung Schürhoff (TMSA). Paratype female: Tanzania, Rungwe Mts, 02.2006, leg. V Kayombo (PCGB).

#### Diagnosis.

This is the only species currently recognised within the newly erected genus. Thus, its diagnostic characters are the same as those highlighted above under the description of *Lophorrhinides* gen. n., in comparison to its sister genus *Lophorrhina*. Within *Lophorrhina*, the species that most closely resembles *Lophorrhinidesmuellerae* sp. n. is *L.donckieri*, but only as far as female superficial characters are concerned. Indeed a photo of the paratype female of *L.muellerae* sp. n. was originally included in a series illustrating that species in the recently published iconographic monograph of Beinhundner (2017: 990, fig. 18). The females of the two species, however, differ remarkably in their clypeal shape, scutellum width/length ratio, body pilosity and general shape, as well as mesometasternal process. As sexual dimorphism is very developed in *Lophorrhina*, but barely recognizable in *Lophorrhinides* gen. n., the males of the two genera are drastically different, aside from their aedeagal shape where some similarities can be observed. The key differences between the two genera can be assessed through the comprehensive set of high quality images, illustrating all the diagnostic characters of three species of the genus *Lophorrhina*, i.e. *L.heinkeli* Beinhundner, 2015, *L.macularia* (Bates, 1888), *L.rigouti* (Allard, 1985), published recently by Beinhundner (2015: pls IV, V).

#### Derivatio nominis

This species is dedicated to Ruth Müller, Senior Curator at the Ditsong Museum of Natural History (Pretoria, formerly Transvaal Museum), who has a long-term record of collaboration with the first author. In 2017, she sent us the male holotype here described, along with other cetoniines currently under study for identification, with the belief that they may represent taxa yet unknown to science.

#### Description of male holotype

*Size*. Length 17.9 mm; width 9.8 mm.

**Figure 1.**
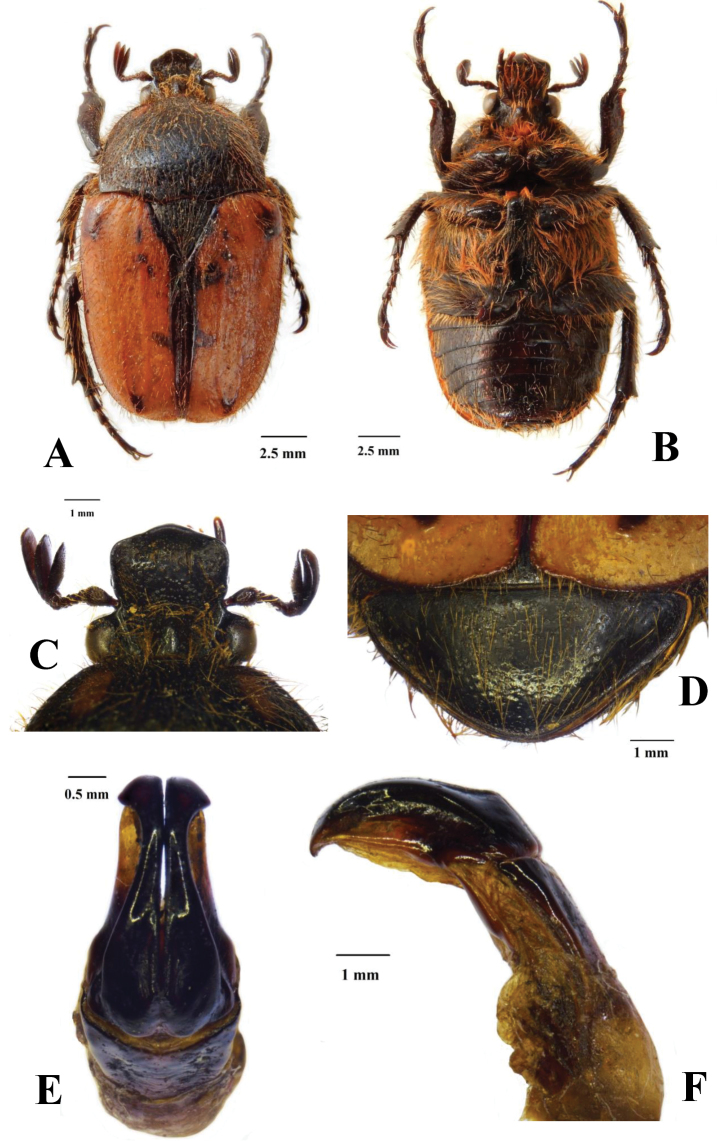
Dorsal (**A**) and ventral (**B**) habitus of male *Lophorrhinidesmuellerae* gen. et sp. n., with details of clypeus (**C**), pygidium (**D**) and aedeagus in dorsal (**E**) and lateral (**F**) view. Photographs Lynette Clennell.

*Body*: Black and ochraceous, without cretaceous markings; black areas shiny, but otherwise matte to velutinous; fine to ultrafine punctures covering virtually entire surface, with yellow to brown, long to very long setae emerging at centre of each puncture (Fig. 1A).

*Head*. Completely black and shiny; clypeus widening anteriorly, deeply concave and sharply upturned at anterior margin to form a horn-like protuberance at middle (Fig. 1C); lateral angles smoothly rounded and clypeo-lateral margins vertically declivous; presence of prominent tubercle at centre of vertex, between supra-ocular tubercles; surface covered in fine and scattered punctures, with yellow-orange setae emerging at centre of most punctures and becoming particularly long towards vertex (Fig. 1C); antenna dark brown, with club slightly longer than flagellum; pedicel dark brown with lighter head attachment and bearing clusters of long, erect yellowish setae.

*Pronotum*. Black and shiny, with two symmetric ochraceous and oblong maculae on each anterior margin of disc; with numerous but well-spaced fine punctures and long straw-coloured setae emerging at centre of punctures; shape semicircular to hexagonal with lateral margins perfectly rounded; antero-lateral margins smoothly rounded, postero-lateral with pronounced angle; posterior margin strongly sinuate with pre-scutellar arch smooth (Fig. 1A).

*Scutellum.* Completely black and shiny; exhibiting identical sculpture and pubescence as pronotum; equilateral triangular in shape with sharp apex; lateral grooves shallow and poorly defined (Fig. 1A).

*Elytron*. Matte to velutinous; ochraceous to orange, with black sutural margin and dark brown to black maculae on humeral and apical callus as well as on upper and central parts of disc, adjacent to sutural margin; costae barely developed and virtually obsolete; sub-humeral arch with very weak sinuation; humeral and apical calluses prominent and with distinct colouration; ultrafine punctures regularly spaced across entire surface, with medium length and erect brown setae emerging at centre of most punctures; apical margin smoothly rounded, without any signs of proximal spines/protuberances; apical and postero-lateral declivities deep but smooth, imparting rather compact and globose body shape (Fig. 1A).

*Pygidium*. Triangular in shape, with very wide base; slightly convex; completely black and covered in regularly spaced horseshoe sculpture; fine but long yellow setae scattered throughout surface (Fig. 1D).

*Legs*. Black and robust, with tarsal segments moderately elongate, with apical tarsal segments at least twice as long as preceding ones; protibia laterally expanded and tridentate, but with second and third teeth virtually obsolete; with longitudinal lines of fine to round punctures and short yellow setae on inner margin; meso- and metatibia with longer and denser yellow setae, densely sculptured and with mid spine on outer carina sharp or moderately developed, respectively; spurs moderately long, slender and acuminate, approximately twice as long in metatibia than in mesotibia (Fig. 1A, B).

*Ventral surface*. Black to dark brown and shiny; with ultrafine sculpture scattered throughout surface, less dense on mesometasternal lobe and on central area of abdominal sternites; with dense pubescence consisting of long yellow to orange setae, shorter and scattered on abdomen and absent on mesometasternal lobe; mesosternal lobe smoothly rounded and not expanded anteriorly or laterally; abdominal sternites with visible concavity at centre (Fig. 1B).

*Aedeagus*. Parameres compact and not particularly elongate; dorsal lobes drastically narrowing anteriorly, with longitudinal groove depression towards mid length and expanding then at apex to form triangular protrusion on each side; apex smoothly rounded and bearing very short scattered setae at margin (Fig. 1E, F); ventral lobes substantially wider and lighter than dorsal lobes (Fig. 1E).

#### Paratype female

*Size*. Length 18.0 mm; width 9.5 mm.

**Figure 2.**
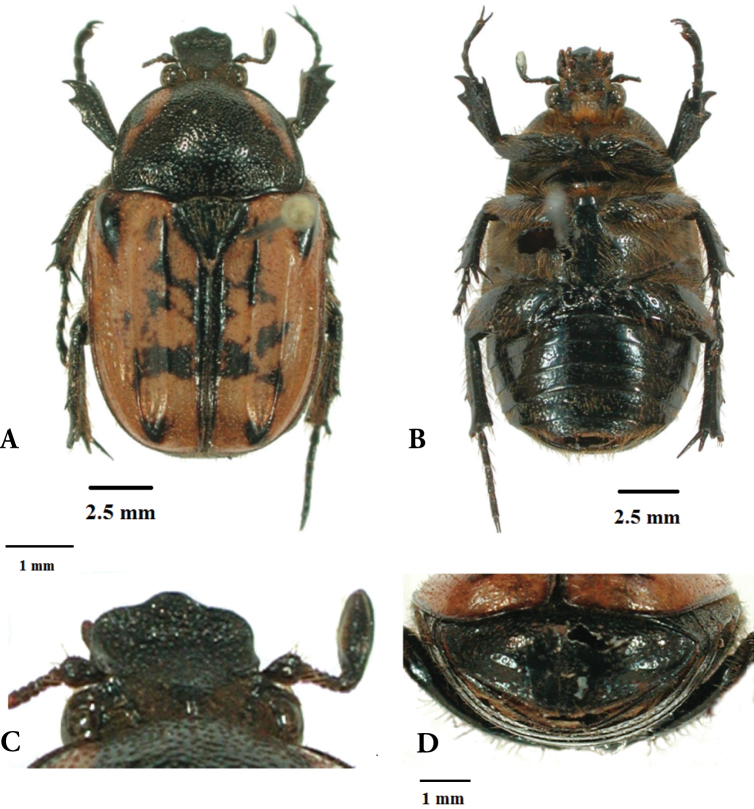
Dorsal (**A**) and ventral (**B**) habitus, with details of clypeal (**C**) and pygidial (**D**) morphology of the female of *Lophorrhinidesmuellerae* gen. et sp. n. Photographs Gerhard Beinhundner.

*Differences to male*. In comparison to the male, the female specimen exhibits a slightly reduced clypeal armature (Fig. 2C), a shinier and markedly less hairy dorsal surface (Fig. 2A), as well as better-defined teeth on all the tibiae. Both elytral and pronotal ornaments are more expanded than in the male, especially the dark maculae on the elytral disc and umbones (Fig. 2A). As in all closely related species, the abdominal segments of the female show a slight ventral convexity (Fig. 2B, D), rather than the typical grooved concavity of its male counterpart.

#### Distribution

**Figure 3.**
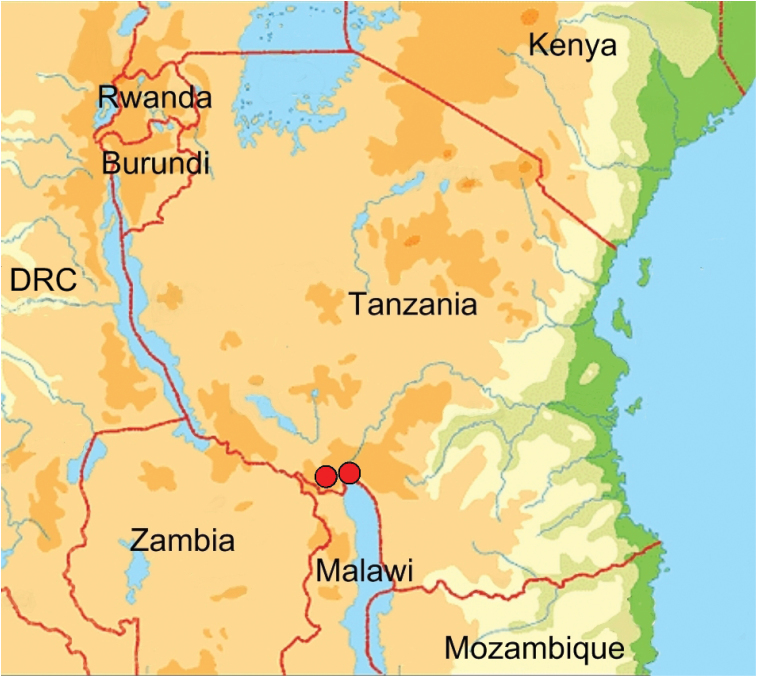
Known geographic distribution of *Lophorrhinidesmuellerae* gen. n. et sp. n. in the southern highlands of Tanzania.

Both known specimens come from the southern highlands of Tanzania, from Manow and Rungwe respectively (Fig. 3). The two localities are approximately 20 km apart at altitudes of 1700–2900 m asl.

## Discussion

The discovery of this new genus and species has come as a surprise, as it clearly represents a novel taxon with at least its male holotype having been available for study in a major museum since its collection in the early 20^th^ century. Interestingly, the only other specimen currently known for this species, a female from Rungwe collected more recently in 2006, had also been overlooked until now and confused with a female of *Lophorrhinadonckieri*, due to a superficial resemblance to that species (cf. Beinhundner 2017: 990, fig. 18). It seems thus likely that other specimens may be “hidden” in other collections around the world. It must also be noted that despite the overwhelming circumstantial evidence in support of the male and female specimens belonging to the same species, there is a margin of doubt regarding this. Molecular phylogenetic analyses could potentially resolve this conclusively, and also help elucidate the relationship with allied genera.

*Lophorrhinidesmuellerae* gen. et sp. n. occurs at the southern end of the distribution range of the *Daedycorrhina* Bates, 1888 group, formerly a separate genus but recently synonymised with *Lophorrhina* by Krajcik (1998). As both specimens appear to originate from high altitude areas in the southern Tanzanian highlands of the East African Rift, it is possible that the genus may represent a geographically isolated relic derived from an ancestral lineage shared with *Lophorrhina* and perhaps other genera like *Anisorrhina* and *Chlorocala*, which show some similarities with *Lophorrhinides* gen. n. especially at the level of the parameres and clypeal or head armature.

Virtually nothing is known about the biology of this new genus and species. Given its close relationship with the Tanzanian and Malawian members of the genus *Lophorrhina*, it is likely that *Lophorrhinides* gen. n. shares some ecological characteristics with species of this group. Unfortunately, even in this case, information on the biology of the various species of *Lophorrhina* that occur in these countries is very scarce, but it seems that virtually all specimens collected were either captured in flight or found “drinking” sap running from the bark of different trees, mostly *Acacia* spp. (Thierry Garnier and Alan J. Gardiner, pers. comm.). However, the high altitude montane habitat where *Lophorrhinidesmuellerae* occurs may suggest a life cycle with a very short life span at the adult stage. This has been shown repeatedly with other genera, particularly in mountainous and/or semiarid environments in southern Africa (e.g., Holm and Marais 1992, Perissinotto et al. 1999, Perissinotto 2017). Here the adults of most cetoniine species emerge from their underground cocoons only after major rainfall events in the late spring or summer and fly and mate during the hottest part of the day. Because they are unable to replenish their energy source through feeding on flowers, sap flows or fermenting fruits, their life span lasts only from several days to a few weeks (Perissinotto et al. 1999).

## Supplementary Material

XML Treatment for Lophorrhinides

XML Treatment for Lophorrhinides muellerae
